# Association of Raynaud’s phenomenon with a polymorphism in the *NOS1* gene

**DOI:** 10.1371/journal.pone.0196279

**Published:** 2018-04-26

**Authors:** Sabrina Munir, Maxim B. Freidin, Susan Brain, Frances M. K. Williams

**Affiliations:** 1 Department of Twin Research and Genetic Epidemiology, King’s College London, London, United Kingdom; 2 Section of Vascular Biology & Inflammation, BHF Centre for Cardiovascular Excellence, School of Cardiovascular Medicine and Sciences, King’s College London, London, United Kingdom; Instituto Nacional de Ciencias Medicas y Nutricion Salvador Zubiran, MEXICO

## Abstract

**Background:**

Raynaud’s phenomenon (RP) describes the phenomenon of recurrent vasospasm of digital arteries, associated with skin colour changes: pallor, cyanosis and erythema. Twin studies have indicated a genetic predisposition for RP; however, the precise aetiology of RP remains unknown. It is thought that genetic variation in temperature-responsive or vasospastic genes might underlie RP so performed a candidate gene study in a large, population based sample. We assessed the association between RP and single nucleotide polymorphisms (SNPs) in the *TRPA1*, *TRPM8*, *CALCA*, *CALCB* and *NOS1* genes.

**Methods:**

Analysis included a total of 4276 individuals from the TwinsUK database. RP status had been determined using validated, self-administered questionnaires and was diagnosed in 640 individuals (17.6%). 66 tag SNPs across the candidate genes were tested for association with RP status using a linear regression model, accounting for covariates. Adjustment was made for multiple testing. RegulomeDB and GTEx databases were used to assess possible functional effects of the polymorphisms.

**Results:**

Nominally significant associations between RP and four SNPs in *NOS1* and one in *CALCB* were identified. After permutation testing, rs527590 SNP in *NOS1* passed the significance threshold. RegulomeDB scores indicated an unlikely functional effect of this variant, while the survey of the GTEx database found the SNP and several variants in linkage disequilibrium to be cis-eQTLs in skin.

**Conclusion:**

Results indicate that RP is associated with variation in gene *NOS1*. This finding may be related to the observation that the significant SNP in *NOS1* is known to exhibit functional influence on the gene expression.

## Introduction

Raynaud’s phenomenon (RP) was first described as “local asphyxia of the extremities” by Maurice Raynaud in 1962 [1]. RP is episodic vasospasm of peripheral arteries, typically affecting fingers but may also affect toes, associated with the characteristic colour changes: pallor (ischemia), cyanosis (deoxygenation) and erythema (reperfusion), often accompanied with pain or paraesthesia (tingling sensation) [2]. RP may be primary—idiopathic or secondary—associated with an underlying, usually rheumatic, condition.

The prevalence of RP is between 3–5% in the general population, of which primary RP accounts for 80–90% [2]. However, the reported prevalence rates vary between different studies depending on the definition used for RP and the population studied [3]. While the aetiology of RP is unknown, there is an associated genetic predisposition as demonstrated by two studies indicating greater concordance amongst monozygotic (MZ) than dizygotic (DZ) twins. Heritability for RP is reported as 55–64% [4, 5].

Vascular tone is normally maintained as a balance between vasoconstrictor and vasodilator tone within the walls of small arteries and arterioles [6]. However, vascular tone is affected by normal homeostatic mechanisms involved in temperature and blood flow regulation with complex interactions. Disruption to the fine balance between vasoconstriction and vasodilation in favour of vasoconstriction results in RP, either via downregulated vasodilation or increased vasoconstriction, usually as a result of factors at the neuronal or endothelial level. Temperature sensing receptor channels named thermo-sensitive transient receptor potential (TRP) ion channels include *TRPA1* and *TRPM8* which are cold sensing and have been suggested to mediate cold-induced vascular responses in skin in vivo, linked at least in part to their expression on perivascular sensory nerves [7]. Neurogenic vasoactive peptides include calcitonin gene related peptide (CGRP) and substance P, which are commonly localised to perivascular sensory nerves [8] and neuronally derived nitric oxide (NO) [9]. Variation in genes encoding these ion channels or vasoactive agents may be expected to impact the balance between vasoconstriction and vasodilation and result in the RP phenotype. To date, reliable genetic associations in a large, population-based sample characterised for RP have not been described. This study, therefore, represents the first attempt to determine association of candidate genes in a large population sample assessed for RP.

Two previous genetic studies have been conducted to identify disease susceptibility regions in RP. A genome-wide linkage screen of 6 families identified 5 areas of possible linkage (*P* value ≤ 0.05), which were associated with the genes encoding the beta subunit of the muscle acetylcholine receptor and the serotonin 1B and 1E receptors [10]. Association of the beta subunit of the muscle acetylcholine receptor is plausible owing to its involvement in vascular tone. Although the association of serotonin 1B (5-HT_1B_) and serotonin 1E (5-HT_1E_) receptors is questionable because they are encoded by genes on chromosome 6, outside the region of linkage, these two genes are biologically plausible due to their vasoactive function. The role of serotonin in the pathophysiology of RP remains unclear. However small studies have reported a decrease in frequency, duration and symptoms of RP following selective serotonin 5HT2 receptor treatment suggesting serotonin in fact has a role in RP [11, 12]. A candidate gene study in a clinical sample examined 4 vasoactive mediator genes (reporting no significant differences in allele frequencies [13]. RP had been diagnosed using validated questionnaires and colour charts as part of a full clinical assessment, but the sample included only 95 primary RP cases.

The recent and growing recognition of cold responsive ion channels informed this work and provided candidate genes for consideration, as well as known neuronal vasodilators. *TRPA1*, *TRPM8*, *CALCA*, *CALCB* and *NOS1* were selected because of their known role in cold induced vascular responses. In murine models, *TRPA1* acts as a primary vascular cold sensor, mediating both initial vasoconstriction and subsequent restorative blood flow, upon paw exposure to cold [7]. A study assessing cold induced responses found that *TRPM8* mediates cold induced autonomic heat gain responses in a systemic model [14]. CGRP is a vasodilator neuropeptide which exists in two isoforms in humans, α-CGRP and β-CGRP, encoded by the genes *CALCA* and *CALCB* respectively [8]. Lastly, the involvement of neuronal nitric oxide synthase (nNOS) derived NO in mediating the restorative vasodilator response after cold treatment [7], provided rationale for investigating the gene *NOS1* encoding nNOS.

The precise aetiology of RP remains unknown and genetic variation in temperature-responsive or vasospastic genes might play a role. The aim of our work was to assess the association between RP and single nucleotide polymorphisms (SNPs) in genes *TRPA1*, *TRPM8*, *CALCA*, *CALCB* and *NOS1*.

## Methods

### Ethics statement

Ethical approval for was obtained from the St Thomas’ Hospital Research Ethics Committee and all twins provided informed consent.

### Study population

Subjects for this study were twins enrolled in the NIHR BRC BioResource TwinsUK adult twin registry based at King’s College London [15]. Sets of monozygotic (MZ) and dizygotic (DZ) same sex Northern European twin pairs have been recruited through successive media campaigns since 1993. For historic reasons, most twins are female. Zygosity was determined using a standard questionnaire and where there was uncertainty, zygosity was confirmed by multiplex DNA fingerprint testing and, more recently, genetic association data.

### Assessment of Raynaud’s phenomenon

All volunteers receive regular questionnaires for self-completion. Questions regarding RP were sent out to the twins as part of larger questionnaires, regarding a range of health and lifestyle issues between 1996 and 2001. Questions were organised so that the hypothesis being tested was not apparent to the respondent. Primary and secondary RP were not distinguished. Classification of RP was based on validated criteria [16]. RP was classified by reporting a history of unusual digital sensitivity to cold and two or more colour changes (white, blue, purple, red, or other). RP status was defined categorically as present or absent using this definition. We have used this method of diagnosing RP in other published genetic studies [4, 17]; and similarly other studies have used responses from questionnaires to diagnose RP [3, 18].

### Selection of single nucleotide polymorphisms (SNPs) genotyped

To minimise multiple testing, we chose tag SNPs accounting for most variation in the genes of interest. The LD TAG SNP Selection tool (https://snpinfo.niehs.nih.gov/snpinfo/snptag.html) was used to identify tagSNPs which were independent of one another (*R*^2^ < 0.65) using a European ancestral population (CEU), in all five candidate genes. A total of 66 tag SNPs were identified: 9 for *TRPA1*, 35 for *TRPM8*, 1 for *CALCA*, 3 for *CALCB* and 18 for *NOS1*. SNP genotypes were coded as 0, 1, and 2 for homozygous wild-type, heterozygous, and homozygous variant, respectively. Minor allele frequency for all SNPs was above 5%.

### Statistical analysis

Statistical analysis was performed using R statistical software with the package GenABEL [19]. We used linear regression model to analyse association between RP and the SNPs accounting for age, sex and the kinship shared by twins in a pair. The *p*-values were obtained through experiment-wise permutation testing (n = 200) and the significance threshold was set at *p* = 0.05.

### Functional assessment of SNPs

Possible functional effects of those SNPs identified as statistically significantly associated with RP were assessed using RegulomeDB (http://www.regulomedb.org/). This database allows assessment of SNPs in non-coding and intergenic regions for functional effects using known and predicted regulatory elements. The RegulomeDB provides scores which refer to the data available for each individual SNP, with lower scores associated with a wider range of supporting data for functional importance. The effects of RP-associated SNPs on gene expression was assessed using GTEx database of expression quantitative trait loci (eQTL) variants, which helps explain the biological effects of genetic variants (http://www.gtexportal.org).

## Results

A total of 5,654 individuals responded to the Raynaud’s phenotyping questionnaires, for which genotype data were available for 4,276 subjects. The final sample comprised 311 MZ pairs, 1,246 DZ pairs and 1,162 singletons of Northern European ancestry (Table 1).

**Table 1:** Characteristics of sample.

Trait		RP positive (n = 640)	RP negative (n = 3636)	Total (n = 4276)
Age, median (range) years		49 (18–76)	51 (18–81)	50 (18–81)
Sex, n (%)	Male	9 (1.41)	297 (8.16)	306 (7.2)
	Female	631 (96.6)	3339 (91.8)	3970 (92.8)
Zygosity, n	MZ	106	516	622
	DZ	371	2121	2492
	Singleton	163	999	1162

RP = Raynaud’s phenomenon; n = number; MZ = monozygotic; DZ = dizygotic.

A total of 640 individuals were classified as RP positive, consisting of 106 MZ individuals, 371 DZ individuals and 163 singletons giving a prevalence of RP in the sample of 15.0%, with a slightly higher prevalence reported amongst MZ subjects (17.0%; not significant compared to the whole sample: Wilcoxon test: p = 0.313). The median age of the sample was 50 (18–81) years. The majority of the sample was female (92.8%).

A total of 66 SNPs from the five candidate genes were analysed for association with RP in the sample including: 9 SNPs across *TRPA1*, 35 across *TRPM8*, 1 across *CALCA*, *3* across *CALCB* and 18 across *NOS1*.

Association analysis showed nominally statistically significant association between RP and 5 SNPs (Table 2) comprising four SNPs in *NOS1* gene, rs527590, rs693534, rs545654, and rs1123425 (p = 0.001, 0.002, 0.004, and 0.005, respectively) and one SNP in *CALCB* gene, rs16930880 (p = 0.002). After applying experiment-wise permutations (n = 200), rs527590 from *NOS1* remained statistically significantly associated with RP (permutation p-value = 0.040).

**Table 2:** Single nucleotide polymorphisms statistically significantly associated with RP.

SNP	Gene	N	MAF	β	SE	Raw p-value	Experiment-wise permutation based p-value
rs527590	*NOS1*	4276	0.203	0.032	0.009	0.001	0.040
rs1123425	*NOS1*	4276	0.473	0.023	0.008	0.002	0.120
rs545654	*NOS1*	4276	0.482	0.021	0.008	0.004	0.205
rs693534	*NOS1*	4276	0.348	-0.022	0.008	0.005	0.275
rs16930880	*CALCB*	4089	0.072	0.044	0.015	0.002	0.120

A linear regression model was used to analyse association between RP and the SNPs accounting for age, sex and the kinship shared by twins in a pair. The experiment-wise permutation p value was based on 200 permutations. β = effect size; SE = standard error; SNP = single nucleotide polymorphism; N = number of genotypes analysed; MAF = minor allele frequency.

The significant rs527590 variant in *NOS1* after permutation testing was evaluated for possible functional effect using RegulomeDB. The variant had a RegulomeDB score of 6 which is classified as “minimal binding evidence”. The results of this analysis suggest lack of proof for functional importance of the SNP found to be associated with RP.

Using GTEx database, the rs527590 and nearby SNPs in linkage disequilibrium were assessed for association with cis-gene expression. Five SNPs of *NOS1* gene were identified as cis-eQTLs in skin and oesophagus mucosa (Table 3).

**Table 3:** Association between *NOS1* gene expression and its SNPs associated with RP.

SNP	Effect Size	p-value	Tissue
rs527590	0.79	2.7 x 10^−16^	Skin—Not Sun Exposed (Suprapubic)
rs527590	0.84	2.7 x 10^−37^	Skin—Sun Exposed (Lower leg)
rs527590	0.37	7.8 x 10^−8^	Oesophagus Mucosa
rs482555	0.82	3.1 x 10^−36^	Skin—Sun Exposed (Lower leg)
rs482555	0.76	2.5 x 10^−15^	Skin—Not Sun Exposed (Suprapubic)
rs482555	0.37	7.8 x 10^−8^	Oesophagus Mucosa
rs3782221	-0.74	1.3 x 10^−30^	Skin—Sun Exposed (Lower leg)
rs3782221	-0.67	3.3 x 10^−13^	Skin—Not Sun Exposed (Suprapubic)
rs4766845	-0.88	1.3 x 10^−39^	Skin—Sun Exposed (Lower leg)
rs4766845	-0.82	7.1 x 10^−17^	Skin—Not Sun Exposed (Suprapubic)
rs4767533	-0.73	1.2 x 10^−29^	Skin—Sun Exposed (Lower leg)
rs4767533	-0.65	5.3 x 10^−13^	Skin—Not Sun Exposed (Suprapubic)

Using GTEx database (https://www.gtexportal.org/home/), the statistically significant tagSNP (rs527590) and those in linkage disequilibrium were assessed for association with cis-gene expression. SNP = single nucleotide polymorphism.

## Discussion

While genome-wide association studies (GWAS) have revolutionised the study of the genetic architecture of common complex traits, they require large samples and to date a GWAS of RP has not been published. Intrigued by recent advances in the understanding of ion channels mediating cold sensitivity, we postulated that variation in the genes encoding such ion channels play a role, either directly or via downstream signalling in the aberrant vascular tone seen in RP. While candidate gene studies have been performed in small clinical samples, the selection of controls is problematic and may lead to covert population stratification which can cause false positive findings.

We have performed the first large scale candidate gene study in the general population. Within TwinsUK there was a reasonable prevalence of RP (15.0%), similar to the UK general population [2], and in keeping with findings in other studies: the prevalence of RP has been reported as high as 21% in women in general practice in the UK [18]. The reported prevalence may vary between studies due to variations in the populations studied as well as the definition used in classifying RP.

We tested 66 tag SNPs in five biologically plausible candidate genes and found 1 variant significantly associated with RP in the study group (Table 2). Permutation based p-value = 0.040 was observed for rs527590 from *NOS1* and passed the significance threshold. The SNP was found to lie within an intronic region and unlikely to possess any functional significance according to the RegulomeDB. GTEx results for this SNP is not known to be associated with gene expression in the most relevant tissue (vascular or peripheral nerve); however, the SNP as well as the variants in LD with it are known as eQTLs in skin tissue and oesophageal mucosa (Table 3). To increase the power of the study by reducing the number of multiple tests and also to ensure the independence of the tests, we chose the strategy to analyse tag SNPs only. Of note, we chose tagSNPs for the study to cover a gene area and not according to functional relevance. Any of the SNPs in the haplotypic blocks not studied directly may potentially account for the association. Although the significantly associated SNP does not appear functional, at least three SNPs that we did not study directly are known to be cis-eQTLs for *NOS1* in relevant tissue (skin), thus suggesting their potential importance for RP. From the results of the current study we can conclude that the variant rs527590 from the *NOS1* gene is associated with RP in the general population and has been found to influence *NOS1* gene expression.

The gene *eNOS* (also known as *NOS3*) was previously included in a candidate gene study but no differences in allele frequencies between patients with primary RP and RP negative matched controls was found [13]. RP was diagnosed by a positive response to both a previously validated questionnaire regarding cold sensitivity and digital colour changes [16], similar to the present study, and Maricq’s colour charts [20]. Patients were then classified as having primary RP if they fulfilled LeRoy’s proposed criteria for primary RP [21]. The sample size in this study was relatively small (95 patients with primary RP, 97 controls) compared to the present study, therefore may be underpowered to detect any statistical significance if variation in the gene is of small effect size. *NOS1* and *eNOS* are two different genes encoding different enzymes with similar functions that are expressed in different tissues, so this can explain the difference in the results.

There are a few limitations in the present study. Based on responses from self-administered questionnaires, it has the possibility of introducing recall bias. However, the twins were not aware of the hypothesis being tested and are accustomed to being studied, with no evidence of recall bias in other studies. Secondly, individuals were not sub-classified as primary or secondary RP so there may be other genetically mediated disease influencing factors—such that genetic variants associated with systemic sclerosis [6]. However, this condition is very rare in the population so not likely to have influenced the overall results. The prevalence of rheumatoid arthritis in our sample is consistent with other population reports (approximately 1%) and we have only a few twins reporting more serious rheumatic conditions so secondary RP is unlikely to be a significant finding in our sample. Information regarding occupational exposure, in particular vinyl chloride monomer (VCM) and vibration, were not available. Studies have investigated the genetic component in the occurrence of RP secondary. One study identified associations between *GST M1* and *GST T1* gene polymorphisms and RP in VCM exposed male subjects [22]. In another study polymorphic variants in the HTR1B gene were associated with the susceptibility of secondary RP in vibration-exposed occupational Chinese Han people [23]. The role of occupational exposure is unlikely to be significant due to few males in our sample. International consensus on the diagnostic criteria for RP was reached, prior to the 9^th^ International Congress on Autoimmunity [24]. It was agreed that biphasic or triphasic colour changes, with white and blue colour changes as the most important colours and a history of cold temperatures as a trigger for RP were required to make a diagnosis of RP. The use of colour charts or cold challenge testing in making a diagnosis was deemed inappropriate. The requirement for clinical assessment still remains debatable due to disagreement amongst the panel. The present study fulfilled the diagnostic criteria outlined as cold sensitivity and a minimum of two reported colour changes were required to classify patients as RP positive. The advantage of questionnaire data is the very large sample, and the fact that a population sample means the results can be extrapolated to the general population of the UK [15].

## Conclusion

In summary, this study has identified one polymorphic variant within the *NOS1* gene as significantly associated with RP in the general population. Even though the functional effect of the variant is questionable according to the RegulomeDB scores, the association of the variant with *NOS1* gene expression in skin is important and should be investigated further with respect to RP. Of note, despite the sensitivity of the cold sensing TRP channels and regulatory activity of CGRP in RP, no significant association was obtained with these genes. The results of this candidate gene study may help focus further study into the *NOS1* gene to identify functional variants responsible for association with RP, and may provide mechanistic information to discover novel therapeutic targets.
